# Validation of Modaplex *POLE* mutation assay in endometrial carcinoma

**DOI:** 10.1007/s00428-023-03636-0

**Published:** 2023-10-24

**Authors:** Eduard Dorca, Ana Velasco, Mar Varela, Sonia Gatius, Sergio Villatoro, Neus Fullana, Dolors Cuevas, Marta Vaquero, Astrid Birnbaum, Karsten Neumann, Xavier Matias-Guiu

**Affiliations:** 1 Pathology Department, Hospital Universitari de Bellvitge, Universitat de Barcelona, IDIBELL, Feixa Llarga SN, 08907 L’Hospitalet de Llobregat, Spain; 2 Pathology Department, Hospital Universitari Arnau de Vilanova, Universitat de Lleida, IRBLleida, CIBERONC, Av Rovira Roure 80, 25198 Lleida, Spain; 3 Städtisches Klinikum Dessau Institut für Pathologie, Dessau-Roßlau, Germany

**Keywords:** Endometrial carcinoma, *POLE* mutation, Molecular classification, Biotype

## Abstract

The TCGA-based molecular classification of endometrial cancer has emerged as an important tool to stratify patients according to prognosis. A simplified scheme has been proposed, by using immunohistochemistry for p53, MSH6, and PMS2 and a molecular test for *POLE* mutations (NGS or Sanger sequencing, techniques that are not available in many centers worldwide). In this study, we validate a novel method that allows simultaneous analysis of multiple pathogenic *POLE* mutations. The Modaplex technology integrates polymerase chain reaction and capillary electrophoresis. The design of this study encompassed 4 different steps: (1) a retrospective-pilot phase, with 80 tumors, balancing the four molecular subgroups. (2) A retrospective phase of 25 tumors obtained between 2016 and 2020, and 30 tumors obtained between 2000 and 2015. (3) An inter-laboratory corssavalidation step with 19 cases (belonging to phases 1 and 2). (4) A prospective cohort of 123 tumors, of unknown *POLE* status, with simultaneous validation by Sanger sequencing. A total of 258 samples were analyzed. In the first and second phases, the test showed positive/negative predictive values of 100%, by correctly identifying *POLE* mutation status in 79/79 and 55/55 cases. Phase 3 showed 100% of inter-laboratory consistency. Phase 4 showed 16 positive samples out of the 123 prospective cases. Overall, the test has revealed sensitivity and specificity of 100%, identifying a total of 47 *POLE*-mutated tumors. We have shown that this technique allows faster and easier identification of multiple pathogenic *POLE* mutations with high robustness and confidence when comparing to other tests such as Sanger sequencing.

**Supplementary information:**

The online version contains supplementary material available at 10.1007/s00428-023-03636-0.

## Introduction

The Cancer Genome Atlas Research Network (TCGA) performed an integrating genomic, transcriptomic, and proteomic characterization of endometrial carcinoma (EC) [1], based on array and sequencing analysis of 373 cases. Exome sequence analysis revealed four groups of tumors. Group 1 included tumors with a very high mutation rate and characteristically inactivating somatic mutations in *POLE* exonuclease. Group 2 was characterized by mismatch repair (MMR) deficiency, and high mutation rates. Group 3 included MMR proficient/*POLE* wild-type tumors with low copy number alterations. Finally, group 4 showed frequent *TP53* mutations and high copy number alterations, associated with a low mutation rate. Prognosis was excellent for group 1, intermediate for groups 2 and 3 with similar progression-free survival rates, and unfavorable for group 4 [2, 3].

The excellent prognosis of the group of *POLE*-mutated tumors has been validated in numerous series of cases [2–11], and has led to incorporation of *POLE* mutation testing in tumor stratification in international guidelines, such as by ESGO-ESTRO-ESP [12] and FIGO [13].

*POLE* mutation testing is usually performed by next-generation sequencing (NGS) or Sanger sequencing, which are regularly performed in the setting of academic or tertiary centers. However, there are difficulties to incorporate *POLE* mutation testing in clinical practice, since these techniques are not available in numerous centers worldwide.

## Materials and methods

### Tissue sample collection

A total of 258 formalin-fixed, paraffin-embedded (FFPE) tissue samples of EC were obtained from the surgical pathology files of the Departments of Pathology in Hospital Universitari Arnau de Vilanova, Lleida, and Hospital Universitari de Bellvitge, L’Hospitalet-Barcelona, Spain. The study was approved by the local ethics committee (Ref PR047/18), February 22, 2018, according to the Declaration of Helsinki and patients signed an informed consent. The main clinical and pathologic data are shown in Supplementary Table 1.

From each case, a FFPE block with adequate viability and tumor representation (of at least 20%) was selected. From each case, eight sections of 10 µm thickness were obtained, subsequently performing DNA isolation using the “Cobas DNA Sample Preparation Kit” and checking DNA quality by Qubit. Cases revealing a DNA concentration under 10 ng/µL were excluded while those exceeding the marked concentration threshold were tested by Modaplex technology.

### Modaplex *POLE/POLD1* Mutation Analysis Kit assay

The combination of PCR and fragment length analysis by automated sequential capillary gel electrophoresis (CE) was performed using a benchtop molecular genetic system (Modaplex TM, BIOTYPE GmbH, Dresden, Germany).

After each PCR cycle, the fluorescently labeled amplicons are electrokinetically injected into the capillary gel. While the PCR reaction continues undisturbed, the injected PCR products are size separated from each other in the gel and detected by laser adsorption. The combination of size separation and detection after each PCR cycle generates real-time data that allows quantification of molecular targets in one PCR reaction. Based on the manufacturer’s instructions for the use of a *POLE/POLD1* mutation assay (Modaplex *POLE/POLD1* Mutation Analysis Kit, BIOTYPE GmbH, Dresden, Germany), a PCR-based multiplex assay detecting 19 single nucleotide mutations within the exonuclease domain of the *POLE* and *POLD1* genes in human DNA from formalin-fixed paraffin-embedded (FFPE) material was performed. The tests allow identification of 9 of the 11 mutations of the catalog of pathogenic *POLE* mutations (P286R, V411L, S297A, S459F, A456P, F367S, L424V, P436R, M444K); see Supplementary Tables 2 and 3 (143).

An aliquot 20 μL of the *POLE/POLD1* master mix was transferred in the corresponding PCR plate. The following reagents are added to the corresponding wells at 5 µL each: extracted and prediluted DNA (input optimum 4 ng) for samples, nuclease-free water to the negative control and *POLE/POLD1* positive control to the positive control.

When using the benchtop molecular genetic system, half of the PCR plate (48 wells) can be used. Empty wells, which do not contain sample or control, must be covered with a diluted buffer (according to the instructions for use).

One PCR reaction contains three calibration templates and their corresponding PCR primer pairs. The calibrator control templates are present at different (pre-set) concentrations and sizes. At the end of the run, the amplified PCR products are sized using the calibrators as markers and quantified using relative Ct value of the calibrators and target(s).

The PCR plate was sealed with an aluminum sealing foil and gently vortexed and spun in a tabletop centrifuge. Afterwards, the seal was removed, and all 48 wells of the PCR plate were covered with a drop of mineral oil. The PCR plate was then sealed again with aluminum foil and gently spun together with a CE plate in a tabletop centrifuge. For generating an electrical current required for initiating capillary electrophoresis, each CE plate contains a medium. The CE module moves and immerses capillaries in CE buffer, where CE separation under applied voltage was performed. Meanwhile, the PCR reaction continues undisturbed. PCR cycling and CE separation are timed to match one complete CE separation within two PCR cycles.

The PCR plate and CE plate were transferred to the instrument. By scanning an assay barcode, assay registration is performed. The barcode contains all relevant information about the PCR and analysis protocol. The device software navigates the user through the start of a run. Amplifications, parallel multiple CE-based separations, and target detections are performed automatically within approximately 3 h. The Modaplex system software automatically determines and analyzes the results. After Ct cut-off for *POLE* mutations based on verification data, the software displays the corresponding mutations.

### Sanger sequencing

DNA from FFPE blocks was purified using the Maxwell FFPE Plus LEV DNA Purification Kit (Promega) according to the manufacturer’s instructions. For all samples, DNA concentration was determined using a Nanodrop ND-1000 spectrophotometer (Thermo Fisher Scientific). The exonuclease domain of *POLE* was screened for mutations by PCR amplification and subsequent Sanger sequencing. The sequenced region included exons 9, 11, 13, and 14. Primer sequences are provided in Supplementary Table 3. PCR products were confirmed by gel electrophoresis and cleaned up with the MinElute PCR Purification Kit (QIAGEN) following the manufacturer’s instructions. Bidirectional Sanger sequencing was performed using the BigDye Terminator v3.1 Cycle Sequencing Kit (Applied Biosystems) and run on a SeqStudio Genetic Analyzer (Applied Biosystems). Variants were identified both manually and automatically in SeqScape analysis software (Life Technologies).

### Design of the study

The design of this study encompasses 4 different phases:

- Phase 1: Pilot phase, composed of 80 ECs balancing molecular subgroups (20 *POLE* mutated, 20 non-specific molecular profile, 20 MMR deficient, 20 p53 abnormal), previously diagnosed in our center and with molecular classification available. *POLE* gene mutational study was previously performed by Sanger sequencing.
- Phase 2: Composed of two different sets of retrospective cases. The first one was composed of 25 cases that were diagnosed between 2016 and 2020. The second one was composed of 30 cases diagnosed between 2000 and 2015. All cases had been subjected to *POLE* Sanger sequencing. The main objective of this second phase was to assess the performance of the test with the aim of checking the test performance using cases with long storage samples with lesser DNA quality. There was an enrichment of grade 3 tumors, to ensure appropriate representation of *POLE*-mutated tumors that accounted for 21.8% of cases (12 samples).
- Phase 3: A selected set of 19 cases of phases 1 and 2 (10 POLE wild-type and 9 *POLE*-mutated tumors) were analyzed by the Modaplex *POLE/POLD1* Mutation Analysis Kit in a different center (Städtisches Klinikum Dessau, Institut für Pathologie, Dessau-Roßlau Germany), to check inter-laboratory consistency of the test.
- Phase 4: Finally, we recruited a prospective cohort, composed of 100 cases diagnosed between 2020 and 2022, in which Modaplex technology was performed simultaneously, and blinded, to the determination of the mutational status of *POLE*, by Sanger.

## Results

A total of 254 samples were finally submitted to the test. Of these, 2 cases were discarded for showing an invalid result.

First phase (Table 1): The test correctly identified 19 of 20 *POLE*-mutated carcinomas and showed a negative result in 60 cases that were previously known to lack a *POLE* mutation. A single false-negative case was found harboring an infrequent double mutation in *POLE* (A456V and A465T), which is not covered by the primer test design, and not included in the current catalog of pathogenic mutations, although one of them (A456V) is located in the same nucleotide of a well-established pathogenic mutation (A456P). This patient also had loss of expression of MSH6, and a germline *MSH6* mutation (c.3261dup). We interpret the case as a patient with Lynch syndrome with two secondary, non-pathogenic *POLE* mutations, not included in the list of mutations detected by the Modaplex *POLE/POLD1* Mutation Analysis Kit. Overall, if we exclude this case, the Modaplex *POLE/POLD1* Mutation Analysis Kit correctly identified *POLE* mutation status in 79 out of 79 cases. Thus, the test showed positive and negative predictive values of 100% and 100%.

**Table 1:**
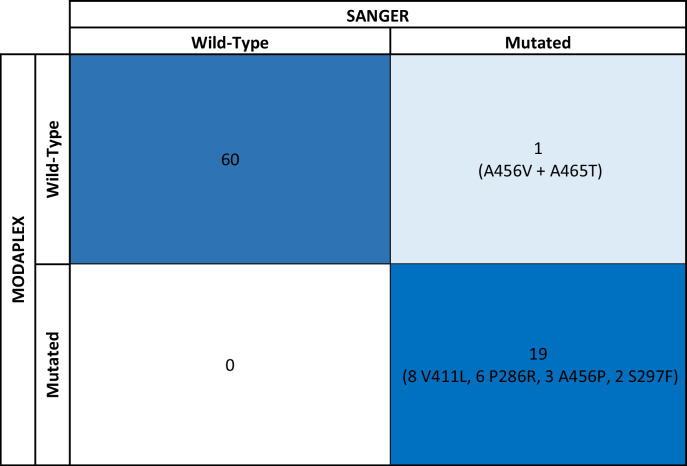
Confusion matrix comparing POLE hotspot mutations detected by Sanger sequencing and Modaplex in the first dataset

Dark blue: True positive and negative results given by MODAPLEX (and confirmed by Sanger sequencing). Light blue: False Negative results (cases detected by Sanger but not by MODAPLEX). White: False Positive results (cases detected by MODAPLEX but not confirmed by Sanger Sequencing)

Second phase (Table 2): In the two sets of a total of 55 retrospective cases, the test detected 12 positive cases and 43 negative cases. The frequency was 21.8%, a little bit higher than expected, probably due to an overrepresentation of grade 3 ECs. There were no differences in results depending on the time the FFPE blocks were stored.

**Table 2:** Comparison between detected mutations by Sanger sequencing and Modaplex in the second/retrospective phase

Manuscript ID	Sample storage time years	POLE Sanger	POLE Modaplex
2.1_1	4	WT	WT
2.1_2	4	WT	WT
2.1_3	4	WT	WT
2.1_4	4	WT	WT
2.1_5	4	WT	WT
2.1_6	4	WT	WT
2.1_7	4	WT	WT
2.1_8	4	WT	WT
2.1_9	4	WT	WT
2.1_10	4	WT	WT
2.1_11	4	WT	WT
2.1_12	3	WT	WT
2.1_13	5	WT	WT
2.1_14	3	WT	WT
2.1_15	2	WT	WT
2.1_16	2	WT	WT
2.1_17	3	WT	WT
2.1_18	5	WT	WT
2.1_19	4	WT	WT
2.1_20	4	WT	WT
2.1_21	4	WT	WT
2.1_22	3	WT	WT
2.1_23	2	L424V	L424V
2.1_24	2	P286R	P286R
2.1_25	5	V411L	V411L
2.2_1	21	WT	WT
2.2_2	16	WT	WT
2.2_3	15	WT	WT
2.2_4	14	WT	WT
2.2_5	14	WT	WT
2.2_6	14	WT	WT
2.2_7	13	WT	WT
2.2_8	12	WT	WT
2.2_9	11	WT	WT
2.2_10	11	WT	WT
2.2_11	10	WT	WT
2.2_12	10	WT	WT
2.2_13	10	WT	WT
2.2_14	9	WT	WT
2.2_15	9	WT	WT
2.2_16	9	WT	WT
2.2_17	9	WT	WT
2.2_18	9	WT	WT
2.2_19	8	V411L	V411L
2.2_20	8	WT	WT
2.2_21	8	V411L	V411L
2.2_22	8	WT	WT
2.2_23	7	WT	WT
2.2_24	22	V411L	V411L
2.2_25	18	P286R	P286R
2.2_26	16	V411L	V411L
2.2_27	17	V411L	V411L
2.2_28	20	P286R	P286R
2.2_29	12	P286R	P286R
2.2_30	18	P286R	P286R

*WT* : Wild Type

Third phase: A total of 19 cases of groups 1 and 2 were selected. Table 3 shows concordance of the results between the two laboratories involved, which reached 100%.

**Table 3:** External validation phase. Reproducibility of Modaplex assay performed in two different centers and confirmation with Sanger sequencing

Manuscript ID	POLE Modaplex Center 1	POLE Modaplex Center 2	POLE Sanger
1_62	A456P	A456P	A456P
1_63	A456P	A456P	A456P
2.1_23	L424V	L424V	L424V
2.1_24	P286R	P286R	P286R
2.2_25	P286R	P286R	P286R
2.1_25	V411L	V411L	V411L
2.2_19	V411L	V411L	V411L
2.2_21	V411L	V411L	V411L
2.2_24	V411L	V411L	V411L
1_33	WT	WT	WT
1_24	WT	WT	WT
1_4	WT	WT	WT
1_28	WT	WT	WT
1_26	WT	WT	WT
1_50	WT	WT	WT
1_27	WT	WT	WT
1_28	WT	WT	WT
1_52	WT	WT	WT
2.1_11	WT	WT	WT
2.1_19	WT	WT	WT
2.1_20	WT	WT	WT
1_53	WT	WT	WT
1_57	WT	WT	WT
1_18	WT	WT	WT
1_19	WT	WT	WT
1_20	WT	WT	WT
2.1_14	WT	WT	WT
2.1_1	WT	WT	WT
2.1_15	WT	WT	WT

*WT*: Wild Type

Fourth phase (Table 4): In this series of 100 prospective cases, the test detected 8 positive samples, representing 8% of cases, within the range of frequency of *POLE*-mutated cases in other series. All of them resulted simultaneously positive by Sanger sequencing.

**Table 4:**
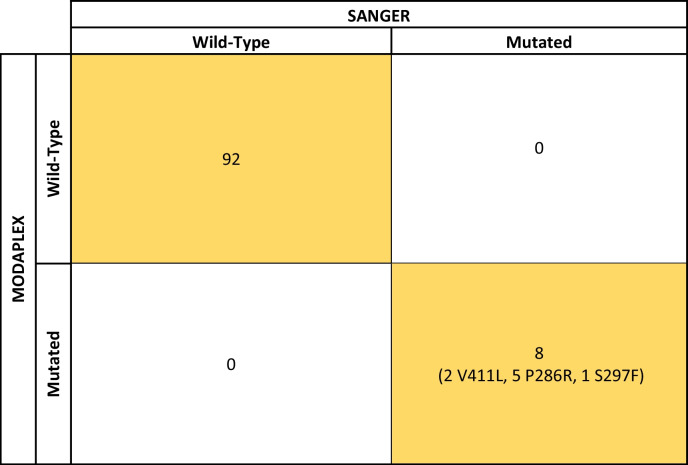
Confusion matrix comparing POLE mutations detected by Sanger sequencing and Modaplex in the prospective cohort

Yellow: True positive and negative results given by MODAPLEX (and confirmed by Sanger Sequencing). White: False positive and negative results (discrepancies between MODAPLEX and Sanger sequencing results)

Overall, the test has revealed sensitivity and specificity values of 100% and 100% respectively for the whole series of 254 valid cases. In this series, the mutations that the test has detected have been the following: 20 V411L, 20 P286R, 3 S297F, 3 A456P, and 1 L424V.

## Discussion

Diagnosis and classification of EC has been based on the microscopic appearance of the tumors [14]. Different histological types have been recognized in the most recent WHO classification: (1) endometrioid carcinoma (EEC), low grade (grades 1 and 2)/high grade (grade 3); (2) serous carcinoma; (3) clear cell carcinoma; (4) mixed carcinoma; (5) undifferentiated carcinoma; (6) carcinosarcoma; (7) neuroendocrine carcinomas; and (8) other unusual types. These diverse histologic types have different molecular features, microscopic appearance, precursor lesions, and natural history. Conventional pathologic analysis remains a very important tool for tumor stratification. Histological typing has, however, problems in inter-observer reproducibility and appropriate assessment of prognosis. While diagnosis is highly reproducible in low-grade EEC, which account for 70% of the cases, and typical serous and clear cell carcinomas, there is poor inter-observer agreement in a 10% of tumors, particularly in a subset of ECs with high-grade morphology. In this scenario, the TCGA-based molecular classification has demonstrated to be a very good tool to assess prognosis, and to establish risk stratification algorithms [5, 6].

At this regard, ESGO-ESTRO-ESP guidelines encouraged to apply the molecular classification in all endometrial cancer patients, particularly in those that have high-grade tumors [12]. Recently published 2023 FIGO guidelines also recommended performing molecular classification in all endometrial carcinomas if possible, specially in stages I and II, where in case of harboring pathogenic mutations on POLE, clinical prognosis remains excellent, and therefore these cases should be downstaged to a IAm. However, there is still some controversy on whether the molecular classification is cost-effective in the group of low-grade tumors [15].

The ProMisE-S algorithm proposed by Talhouk et al. [16] studied cost effectiveness of determining POLE mutations in all EC, defining a “very low risk” EC subgroup in which POLE mutational analysis would not be required (G1/G2, endometrioid, MMR-proficient, p53 wild-type, stage IA, no lymphovascular space invasion). The study concluded that POLE testing should be further restricted to only those patients in whom molecular classification would alter adjuvant therapy recommendations.

*POLE* mutation testing is an important part of the TCGA-based molecular classification, together with immunohistochemical demonstration of the expression of mismatch repair proteins, and p53. *POLE* mutation testing is usually performed by NGS with panels including the exonuclease domain of *POLE*, or by Sanger sequencing of exons 9, 11, 13, and 14 of *POLE*, where the pathogenic mutations are located. While NGS and Sanger sequencing are nowadays regularly performed in reference centers, some institutions around the globe do not have these technologies available, which, in fact, creates a real problem of lack of equity of patients that are treated in different geographical areas. This is important because patients with *POLE*-mutated tumors may be deescalated for adjuvant therapy which are not as effective and may produce side effects [12].

Overall, *POLE*-mutated tumors account for around 10% of ECs. Different studies have confirmed the good prognosis of patients with this type of tumor [2–11]. A recent meta-analysis of 294 patients with tumors with *POLE* pathogenic mutations shows that they occur in younger women (median age 57.0), predominantly at stages I and II, with a tendency to occur in high-grade tumors [17]. Although pathogenic mutations tend to occur in endometrioid tumors, they can be found in any histologic type, including undifferentiated carcinomas or carcinosarcomas. The two most frequent pathogenic mutations are P286R and V411L that account for 80% of the cases. Leon-Castillo reported a catalog of 11 pathogenic mutations, which included P286R, V411L, S297F, S459F, A456P, F367S, L424I, M295R, P436R, M444K, and D368Y, all of them in exons 9, 11, 13, and 14 [18]. Around 9% of *POLE*-mutated tumors are found at the advanced stage (III/IV), and around 4% show progression of disease (30% of those at advanced stages). Around 1% of patients with pathogenic *POLE* mutations died of the tumor. There is a need for simple techniques of *POLE* mutation analysis, able to be incorporated in pathology departments. Modaplex and other tests, such as multiple SNaPshot assay [19], are good opportunities to allow easy routine *POLE* testing in an efficient and cost-effective approach. Appropriate validation process with a significant number of cases is required.

In this study, we tried to validate Modaplex technology in four different cohorts of patients. According to the information provided by manufacturers, the instrument has a Conformité Européenne (CE) marking for electronic devices. In the first retrospective one, we selected 80 tumors, in a way that we examined 20 tumors of each molecular subtype (*POLE*-mutated. MMR deficient, non-specific molecular subtype, p53 abnormal), previously analyzed by immunohistochemistry and *POLE* Sanger sequencing. The Modaplex *POLE/POLD1* Mutation Analysis Kit appropriately identified *POLE* mutational status in 79 out of 80 tumors. The single false-negative case showed an infrequent double mutation in *POLE* (A456V and A465T) which are not covered by the primer test design, and not included in the current catalog of pathogenic mutations, although one of them (A456V) is located in the same nucleotide of a well-established pathogenic mutation (A456P). The case was reinterpreted as a patient with Lynch syndrome, and a *MSH6* germline mutation, and two non-pathogenic *POLE* mutations, not included in the list of mutations identifiable by the test.

In the second retrospective cohort of 55 cases, we wanted to check the test performance in two groups of tumors with different times of storage; one of recent tumors (2016–2020), and one composed of old cases (2000–2015). There was an enrichment of high-grade endometrioid tumors, to ensure a good representation of *POLE*-mutated tumors, which accounted for 21.8% of the cases. It is nowadays very clear that the success for a molecular test in tumor tissue from FFPE blocks depends in part to the impact of what we call “pre-analytical variables” that include inappropriate fixation conditions (type of fixative, delayed fixation, overfixation), but also time and conditions in which the FFPE block of tissue is stored [20–23]. For some biomarkers, including immunohistochemical demonstration of some proteins, the tests do better in “recent” cases in comparison with “old” cases. In this study, time of storage seems not to be influential in the results, since test performance was identical in the two groups.

The third phase consisted in checking interlaboratory performance. A set of 19 tumors from the first two cohorts was selected, and FFPE sections as well as DNA were sent to a second laboratory for screening. There was concordance in 100% of the cases.

Finally, a prospective series of 100 tumors was checked by the Modaplex *POLE/POLD1* Mutation Analysis Kit, with Sanger sequencing validation, in an attempt to assess test performance in a real-world scenario. In this series, the Modaplex *POLE/POLD1* Mutation Analysis Kit appropriately identified 8 positive samples, representing 13% of cases, within the range of frequency of *POLE*-mutated cases in other series.

In view of these results, we can conclude that the Modaplex *POLE/POLD1* Mutation Analysis Kit is a promising technology that allows the determination of the main “Hotspot” mutations in *POLE* gene in a fast, practical, and efficient way. We have shown that this standardized technique allows faster and easier identification of multiple functional *POLE* targets in the context of molecular classification of EC compared to the established method of Sanger sequencing. The main current limitation is that in its present format, the Modaplex *POLE/POLD1* Mutation Analysis Kit identifies 9 out of 11 mutations of the catalog of pathogenic *POLE* mutations. However, the two mutations that are not detected by the test (M295R, and D368Y) account for a very small number of tumors [18].

### Supplementary information

Below is the link to the electronic supplementary material.

Supplementary file1 (DOCX 36 KB)

## Data Availability

This paper that reviews the obtained results from TCGA and COSMIC endometrial cancer datasets, reported a small proportion of this two mutations among the whole cohort of ultramutated endometrial carcinomas.
