# Supplementary material for: Validation of Modaplex POLE mutation assay in endometrial carcinoma
Source: Virchows Arch. 2023 Oct 24;483(6):787–94. doi: 10.1007/s00428-023-03636-0 (PMC10700217; doi:10.1007/s00428-023-03636-0)
Supplement: Supplementary file 1 — Supplementary file1 (DOCX 36 KB) [file 428_2023_3636_MOESM1_ESM.docx]

Supplementary information

**Supplementary Table 1: Demographic and clinical data of the study dataset.**

**NA**: Not available; **EC**: Endometrioid Carcinoma, **SC**: Serous Carcinoma, **CCC**: Clear Cell Carcinoma, **CS**: Carcinosarcoma, **E/S**: Mixed Endometrioid/SC, **UC**: Unidifferentiated Carcinoma; **CNL**: Copy-Number Low (Non specific molecular profile), **CNH**: Copy-Number High (TP53 mutated/Serous-like) , **MSI**: Microsatellite Instabillity (Hypermutated), **POLE**: POLE mutated (Ultramutated).

**Supplementary Table 2: List of primers used for PCR amplification and Sanger sequencing of the POLE exonuclease domain.**

**Supplementary Table 3: Hotspot mutations detected by Modaplex POLE/POLD1 assay.**

**Suplementary Table 1**

| **Manuscript ID** | **Patient-Age** | **Histological classification** | **Grade** | **Molecular classification** | **FIGO Staging 2021** | **POLE**  **Sanger** | **POLE MODAPLEX** |
| --- | --- | --- | --- | --- | --- | --- | --- |
| 1_1 | 60 | EC | 1 | CNL | IA | WT | WT |
| 1_2 | 72 | EC | 1 | CNL | IA | WT | WT |
| 1_3 | 34 | EC | 3 | CNL | IIIA | WT | WT |
| 1_4 | 67 | EC | 3 | CNL | IB | WT | WT |
| 1_5 | 62 | EC | 1 | CNL | IA | WT | WT |
| 1_6 | 69 | EC | 2 | CNL | IIIC1 | WT | WT |
| 1_7 | 50 | EC | 3 | CNL | II | WT | WT |
| 1_8 | 77 | EC | 1 | CNL | IB | WT | WT |
| 1_9 | 63 | EC | 1 | CNL | IA | WT | WT |
| 1_10 | 71 | EC | 1 | CNL | IIIA | WT | WT |
| 1_11 | 61 | EC | 1 | CNL | IA | WT | WT |
| 1_12 | 61 | EC | 3 | CNL | IIIA | WT | WT |
| 1_13 | 79 | EC | 3 | CNL | IA | WT | WT |
| 1_14 | 70 | EC | 3 | CNL | IIIA | WT | WT |
| 1_15 | 79 | EC | 3 | CNL | IIIC1 | WT | WT |
| 1_16 | 74 | EC | 3 | CNL | II | WT | WT |
| 1_17 | 83 | EC | 3 | CNL | IA | WT | WT |
| 1_18 | 65 | EC | 3 | CNL | IA | WT | WT |
| 1_19 | 59 | EC | 3 | CNL | IB | WT | WT |
| 1_20 | 65 | EC | 3 | CNL | IA | WT | WT |
| 1_21 | 68 | EC | 3 | MSI | IB | WT | WT |
| 1_22 | 61 | EC | 3 | MSI | IA | WT | WT |
| 1_23 | 55 | EC | 3 | MSI | II | WT | WT |
| 1_24 | 87 | CCC | 3 | MSI | II | WT | WT |
| 1_25 | 74 | EC | 3 | MSI | IB | WT | WT |
| 1_26 | 74 | EC | 1 | MSI | IA | WT | WT |
| 1_27 | 67 | EC | 3 | MSI | IA | WT | WT |
| 1_28 | 87 | EC | 3 | MSI | IIIC1 | WT | WT |
| 1_29 | 65 | EC | 3 | MSI | IA | WT | WT |
| 1_30 | 57 | EC | 3 | MSI | IA | WT | WT |
| 1_31 | 82 | EC | 3 | MSI | IIIC1 | WT | WT |
| 1_32 | 51 | EC | 3 | MSI | IIIA | WT | WT |
| 1_33 | 87 | EC | 3 | MSI | IIIA | WT | WT |
| 1_34 | 71 | EC | 3 | MSI | IA | WT | WT |
| 1_35 | 58 | EC | 2 | MSI | IVB | WT | WT |
| 1_36 | 69 | EC | 2 | MSI | IA | WT | WT |
| 1_37 | 79 | EC | 3 | MSI | NA | WT | WT |
| 1_38 | 62 | EC | 3 | MSI | IB | WT | WT |
| 1_39 | 60 | EC | 1 | MSI | IA | WT | WT |
| 1_40 | 93 | EC | 3 | MSI | IB | WT | WT |
| 1_41 | 83 | EC | 3 | CNH | IA | WT | WT |
| 1_42 | 76 | SC | 3 | CNH | IVB | WT | WT |
| 1_43 | 58 | CS | 3 | CNH | IA | WT | WT |
| 1_44 | 71 | SC | 3 | CNH | IA | WT | WT |
| 1_45 | 70 | SC | 3 | CNH | IIIC1 | WT | WT |
| 1_46 | 58 | EC | 2 | CNH | IA | WT | WT |
| 1_47 | 64 | SC | 3 | CNH | IA | WT | WT |
| 1_48 | 69 | SC | 3 | CNH | IA | WT | WT |
| 1_49 | 56 | CS | 3 | CNH | IVB | WT | WT |
| 1_50 | 67 | SC | 3 | CNH | IIIA | WT | WT |
| 1_51 | 77 | SC | 3 | CNH | IB | WT | WT |
| 1_52 | 88 | EC | 3 | CNH | IIIB | WT | WT |
| 1_53 | 74 | SC | 3 | CNH | IB | WT | WT |
| 1_54 | 64 | SC | 3 | CNH | IA | WT | WT |
| 1_55 | 63 | EC | 3 | CNH | IA | WT | WT |
| 1_56 | 80 | EC | 3 | CNH | IA | WT | WT |
| 1_57 | 71 | EC | 3 | CNH | IIIC1 | WT | WT |
| 1_58 | 81 | CS | 3 | CNH | IA | WT | WT |
| 1_59 | 63 | EC | 3 | CNH | IA | WT | WT |
| 1_60 | 74 | EC | 3 | CNH | IA | WT | WT |
| 1_61 | 57 | EC | 3 | POLE | IA | V411L | V411L |
| 1_62 | 63 | EC | 3 | POLE | IB | A456P | A456P |
| 1_63 | 61 | EC | 3 | POLE | IA | A456P | A456P |
| 1_64 | 79 | EC | 3 | POLE | IA | V411L + A463V | V411L |
| 1_65 | 63 | EC | 3 | POLE | IA | V411L | V411L |
| 1_66 | 55 | EC | 3 | POLE | IVB | A456P | A456P |
| 1_67 | 57 | E/S | 3 | POLE | IA | P286R | P286R |
| 1_68 | 59 | EC | 3 | POLE | IIIA | P286R | P286R |
| 1_69 | 90 | EC | 3 | POLE | II | P286R | P286R |
| 1_70 | 55 | EC | 1 | POLE | IB | S297F | S297F |
| 1_71 | 71 | EC | 3 | POLE | IA | V411L | V411L |
| 1_72 | 70 | EC | 1 | POLE | IIIA | V411L | V411L |
| 1_73 | 76 | UC | 3 | POLE | IA | V411L | V411L |
| 1_74 | 78 | EC | 3 | POLE | IA | S297F | S297F |
| 1_75 | 578 | EC | 3 | POLE | IC | P286R | P286R |
| 1_76 | 56 | EC | 3 | POLE | IA | V411L | V411L |
| 1_77 | 73 | EC | 3 | POLE | IIIA | P286R | P286R |
| 1_78 | 68 | EC | 3 | POLE | IB | V411L | V411L |
| 1_79 | 56 | EC | 3 | POLE | NA | P286R | P286R |
| 1_80 | 54 | EC | 3 | MSI | IA | A456V+ A465T | WT |
| 2.1_1 | 63 | EC | 2 | CNL | IB | WT | WT |
| 2.1_2 | 76 | EC | 1 | CNL | IA | WT | WT |
| 2.1_3 | 62 | EC | 2 | CNL | IA | WT | WT |
| 2.1_4 | 71 | SC | 3 | CNH | IA | WT | WT |
| 2.1_5 | 79 | CCC | 3 | CNL | IA | WT | WT |
| 2.1_6 | 69 | EC | 1 | CNL | IB | WT | WT |
| 2.1_7 | 61 | EC | 1 | CNL | IB | WT | WT |
| 2.1_8 | 73 | EC | 1 | CNL | IA | WT | WT |
| 2.1_9 | 67 | SC | 3 | CNH | IIIA | WT | WT |
| 2.1_10 | 67 | SC | 3 | CNH | IA | WT | WT |
| 2.1_11 | 71 | SC | 3 | CNH | IA | WT | WT |
| 2.1_12 | 68 | EC | 1 | CNL | IA | WT | WT |
| 2.1_13 | 84 | EC | 3 | CNL | IB | WT | WT |
| 2.1_14 | 89 | EC | 3 | CNL | IVB | WT | WT |
| 2.1_15 | 63 | SC | 3 | CNH | IA | WT | WT |
| 2.1_16 | 78 | SC | 3 | CNH | IIIC2 | WT | WT |
| 2.1_17 | 65 | CS | 3 | CNH | IA | WT | WT |
| 2.1_18 | 75 | SC | 3 | CNH | IVB | WT | WT |
| 2.1_19 | 64 | SC | 3 | CNH | IA | WT | WT |
| 2.1_20 | 66 | SC | 3 | CNH | IA | WT | WT |
| 2.1_21 | 87 | SC | 3 | CNH | IA | WT | WT |
| 2.1_22 | 84 | SC | 3 | CNH | IIIC1 | WT | WT |
| 2.1_23 | 37 | EC | 3 | POLE | IIIC2 | L424V | L424V |
| 2.1_24 | 62 | EC | 3 | POLE | IA | P286R | P286R |
| 2.1_25 | 78 | EC | 3 | POLE | IA | V411L | V411L |
| 2.2_1 | 78 | EC | 3 | CNH | IA | WT | WT |
| 2.2_2 | 60 | EC | 3 | NA | IA | WT | WT |
| 2.2_3 | 50 | EC | 3 | NA | IA | WT | WT |
| 2.2_4 | 61 | EC | 3 | NA | IA | WT | WT |
| 2.2_5 | 65 | EC | 3 | NA | IB | WT | WT |
| 2.2_6 | 61 | EC | 3 | NA | IB | WT | WT |
| 2.2_7 | 74 | EC | 3 | NA | IA | WT | WT |
| 2.2_8 | 67 | EC | 3 | NA | IIIA | WT | WT |
| 2.2_9 | 57 | EC | 3 | NA | IA | WT | WT |
| 2.2_10 | 87 | EC | 3 | NA | IA | WT | WT |
| 2.2_11 | 55 | EC | 3 | NA | IIIB | WT | WT |
| 2.2_12 | 59 | EC | 3 | NA | IB | WT | WT |
| 2.2_13 | 87 | EC | 3 | NA | IA | WT | WT |
| 2.2_14 | 68 | EC | 3 | NA | IA | WT | WT |
| 2.2_15 | 74 | EC | 3 | NA | IA | WT | WT |
| 2.2_16 | 65 | EC | 3 | NA | IIIC1 | WT | WT |
| 2.2_17 | 61 | EC | 3 | NA | IA | WT | WT |
| 2.2_18 | 77 | EC | 3 | MSI | IB | WT | WT |
| 2.2_19 | 79 | EC | 3 | POLE | IA | V411L | V411L |
| 2.2_20 | 79 | EC | 3 | MSI | IB | WT | WT |
| 2.2_21 | 74 | EC | 3 | POLE | IB | V411L | V411L |
| 2.2_22 | 63 | EC | 3 | CNL | IA | WT | WT |
| 2.2_23 | 71 | EC | 3 | MSI | IB | WT | WT |
| 2.2_24 | 56 | EC | 3 | POLE | IB | V411L | V411L |
| 2.2_25 | 83 | EC | 3 | POLE | IVB | P286R | P286R |
| 2.2_26 | 70 | EC | 3 | POLE | IIIC2 | V411L | V411L |
| 2.2_27 | 72 | EC | 3 | POLE | IA | V411L | V411L |
| 2.2_28 | 69 | EC | 3 | POLE | IA | P286R | P286R |
| 2.2_29 | 62 | EC | 3 | POLE | IB | P286R | P286R |
| 2.2_30 | 67 | EC | 3 | POLE | II | P286R | P286R |
| 4_1 | 74 | EC | 3 | CNL | IA | WT | WT |
| 4_2 | 71 | CCC | 3 | CNL | IVB | WT | WT |
| 4_3 | 65 | CS | 3 | CNH | IA | WT | WT |
| 4_4 | 67 | SC | 3 | CNH | IVB | WT | WT |
| 4_5 | 77 | SC | 3 | CNH | IIIC2 | WT | WT |
| 4_6 | 75 | CS | 3 | CNL | IA | WT | WT |
| 4_7 | 82 | SC | 3 | POLE | IA | P286R | P286R |
| 4_8 | 68 | EC | 3 | CNH | IB | WT | WT |
| 4_9 | 82 | EC | 3 | CNL | IB | WT | WT |
| 4_10 | 73 | EC | 3 | MSI | IB | WT | WT |
| 4_11 | 65 | EC | 3 | MSI | IA | WT | WT |
| 4_12 | 82 | EC | 3 | MSI | IIIA | WT | WT |
| 4_13 | 72 | EC | 3 | CNL | IVB | WT | WT |
| 4_14 | 76 | EC | 3 | MSI | IIIC2 | WT | WT |
| 4_15 | 72 | EC | 3 | POLE | IIIA | V411L | V411L |
| 4_16 | 77 | SC | 3 | CNH | IIIA | WT | WT |
| 4_17 | 68 | EC | 3 | MSI | IA | WT | WT |
| 4_18 | 69 | EC | 3 | MSI | IIIC1 | WT | WT |
| 4_19 | 87 | SC | 3 | CNH | IA | WT | WT |
| 4_20 | 83 | EC | 3 | MSI | IIIC1 | WT | WT |
| 4_21 | 89 | SC | 3 | CNH | IB | WT | WT |
| 4_22 | 54 | EC | 3 | CNH | IA | WT | WT |
| 4_23 | 85 | CS | 3 | CNH | IA | WT | WT |
| 4_24 | 75 | EC | 3 | CNL | IB | WT | WT |
| 4_25 | 82 | EC | 3 | CNL | IB | WT | WT |
| 4_26 | 71 | EC | 3 | CNL | IA | WT | WT |
| 4_27 | 82 | EC | 3 | POLE | IA | P286R | P286R |
| 4_28 | 77 | CS | 3 | CNH | IIIC1 | WT | WT |
| 4_29 | 75 | SC | 3 | CNH | IIIC1 | WT | WT |
| 4_30 | 75 | EC | 3 | MSI | IB | WT | WT |
| 4_31 | 62 | EC | 3 | MSI | IA | WT | WT |
| 4_32 | 59 | EC | 3 | MSI | IIIC1 | WT | WT |
| 4_33 | 79 | SC | 3 | CNH | IIIC1 | WT | WT |
| 4_34 | 61 | CS | 3 | CNL | IVB | WT | WT |
| 4_35 | 63 | EC | 3 | MSI | IVB | WT | WT |
| 4_36 | 76 | SC | 3 | CNH | IIIA | WT | WT |
| 4_37 | 83 | EC | 3 | MSI | IIIC1 | WT | WT |
| 4_38 | 73 | EC | 3 | MSI | IA | WT | WT |
| 4_39 | 49 | EC | 2 | CNL | IIIA | WT | WT |
| 4_40 | 86 | EC | 1 | CNL | IB | WT | WT |
| 4_41 | 73 | EC | 1 | MSI | IB | WT | WT |
| 4_42 | 63 | EC | 2 | MSI | IA | WT | WT |
| 4_43 | 58 | EC | 1 | CNL | IA | WT | WT |
| 4_44 | 59 | EC | 2 | MSI | IIIC1 | WT | WT |
| 4_45 | 63 | EC | 1 | MSI | IVB | WT | WT |
| 4_46 | 57 | EC | 2 | MSI | II | WT | WT |
| 4_47 | 73 | EC | 2 | MSI | IA | WT | WT |
| 4_48 | 53 | EC | 1 | CNL | IB | WT | WT |
| 4_49 | 70 | EC | 3 | CNL | IB | WT | WT |
| 4_50 | 62 | EC | 2 | MSI | IB | WT | WT |
| 4_51 | 59 | EC | 1 | CNL | IA | WT | WT |
| 4_52 | 70 | EC | 1 | CNL | IA | WT | WT |
| 4_53 | 66 | EC | 1 | MSI | IA | WT | WT |
| 4_54 | 58 | EC | 3 | CNL | IIIA | WT | WT |
| 4_55 | 74 | EC | 1 | CNL | IB | WT | WT |
| 4_56 | 66 | EC | 1 | CNL | IB | WT | WT |
| 4_57 | 87 | EC | 2 | CNL | IB | WT | WT |
| 4_58 | 68 | EC | 1 | CNL | IB | WT | WT |
| 4_59 | 46 | EC | 2 | CNL | IVB | WT | WT |
| 4_60 | 57 | EC | 2 | MSI | II | WT | WT |
| 4_61 | 65 | EC | 1 | CNL | IVB | WT | WT |
| 4_62 | 68 | EC | 1 | CNL | IA | WT | WT |
| 4_63 | 70 | EC | 2 | CNL | IA | WT | WT |
| 4_64 | 76 | EC | 1 | CNL | IA | WT | WT |
| 4_65 | 70 | EC | 1 | CNL | IB | WT | WT |
| 4_66 | 37 | EC | 2 | MSI | IIIA | WT | WT |
| 4_67 | 70 | EC | 1 | MSI | IA | WT | WT |
| 4_68 | 69 | EC | 1 | MSI | IA | WT | WT |
| 4_69 | 62 | EC | 1 | MSI | IB | WT | WT |
| 4_70 | 62 | UC | 3 | POLE | IV | P286R | P286R |
| 4_71 | NA | EC | 3 | CNL | NA | WT | WT |
| 4_72 | 69 | EC | 2 | MSI | IB | WT | WT |
| 4_73 | 63 | EC | 1 | CNL | IA | WT | WT |
| 4_74 | 34 | EC | 1 | CNL | IA | WT | WT |
| 4_75 | 71 | EC | 1 | CNL | IB | WT | WT |
| 4_76 | 76 | EC | 1 | CNL | IB | WT | WT |
| 4_77 | 81 | EC | 3 | MSI | IB | WT | WT |
| 4_78 | 66 | EC | 2 | CNL | IA | WT | WT |
| 4_79 | 76 | EC | 1 | MSI | IA | WT | WT |
| 4_80 | 57 | EC | 1 | MSI | IA | WT | WT |
| 4_81 | 45 | EC | 2 | MSI | IIIC2 | WT | WT |
| 4_82 | 63 | EC | 1 | CNL | IA | WT | WT |
| 4_83 | 70 | EC | 1 | CNL | 1A | WT | WT |
| 4_84 | 72 | EC | 3 | CNL | IB | WT | WT |
| 4_85 | 70 | EC | 1 | CNL | 1A | WT | WT |
| 4_86 | 74 | EC | 2 | POLE | IB | S297F | S297F |
| 4_87 | 70 | EC | 2 | POLE | IB | P286R | P286R |
| 4_88 | NA | EC | NA | CNL | NA | WT | WT |
| 4_89 | 54 | EC | 1 | POLE | IA | P286R | P286R |
| 4_90 | 73 | EC | 1 | POLE | IA | V411L | V411L |
| 4_95 | 62 | EC | 1 | CNL | IA | WT | WT |
| 4_96 | 74 | EC | 1 | CNL | IA | WT | WT |
| 4_97 | 65 | EC | 2 | CNL | IA | WT | WT |
| 4_98 | 57 | CS | 3 | CNL | IIIC2 | WT | WT |
| 4_99 | 70 | EC | 1 | CNL | IA | WT | WT |
| 4_100 | 84 | EC | 1 | CNL | IA | WT | WT |

**Supplementary Table 2:**

| **Exon** | **Forward  5'-3'** | **Reverse 5'-3'** |
| --- | --- | --- |
| 9 | TGCTTATTTTGTCCCCACAG | TACTTCCCAGAAGCCACCTG |
|  |  |  |
| 11 | GCAGACCTCTGACTGCTGTG | CCTAAGTGCACATGGGAAGC |
|  |  |  |
| 13 | TCTGTTCTCATTCTCCTTCCAG | CGGGATGTGGCTTACGTG |
| 14 | ACCCTGGGCTCTTGATTTTT | CACCTCCATTCAGCTCCAGT |

**Supplementary Table 3:**

| **Gene** | **Mutation** |
| --- | --- |
| **POLE** | T278M |
|  | P286H |
|  | P286R |
|  | P286L |
|  | P286S |
|  | S297A |
|  | S297F |
|  | F367S |
|  | V411L G>C |
|  | V411L G>T |
|  | H422N |
|  | L424V |
|  | P436R |
|  | M444K |
|  | A456P |
|  | S459F |
|  | A465V |
|  | IC1 |
| **POLD1** | D316N |
|  | C319Y |
|  | S478N |
|  | IC2 |
